# Hemodynamic Forces Regulate Embryonic Stem Cell Commitment to Vascular Progenitors

**DOI:** 10.2174/157340308786349471

**Published:** 2008-11

**Authors:** Tzung K Hsiai, Joseph C. Wu

**Affiliations:** 1 Department of Biomedical Engineering and Division of Cardiovascular Medicine, University of Southern California, Los Angeles, CA 90089-1111, USA; 2 Department of Medicine, Division of Cardiovascular Medicine and Department of Radiology, Stanford University, Palo Alto, CA, USA

## Abstract

Pluripotent embryonic stem can (ES) cells can differentiate into all cell lineages. During the process of embryonic development, ES cells are exposed to fluid flow or blood flow generated by the contracting heart. Absence of fluid flow results in the formation of abnormal cardiac chambers and valve formation. Thus, hemodynamic forces and ES cell differentiation to vascular progenitor cells (VPCs) are of emerging interests for restoring endothelial dysfunction, inducing angiogenesis, and forming blood vessel networks. Hemodynamic forces such as fluid shear stress increase the percentage of cells in the S and G_2_-M phases, and induce decondensation of chromatin for gene transcription. Fluid shear stress further accelerates ES commitment to CD31^+^ VPC vascular progenitor cells. These ES-derived CD31^+^ cells express endothelial nitric oxide synthase (eNOS) and von Willebrand factor (vWF). They are also capable of LDL uptake and tubular network formation. In this context, understanding hemodynamic forces and ES cell kinetics of differentiation towards endothelial lineage has potential therapeutic applications for repairing vascular damage and engineering vascular graft. Multidisciplinary team approach will likely garner momentum and synergize expertise to address the current road blocks in basic stem cell research for engraftable, restorative, low immunogenic, and non-tumorigenic endothelial progenitors in high purity and stability.

## INTRODUCTION

**Fig. (1):**
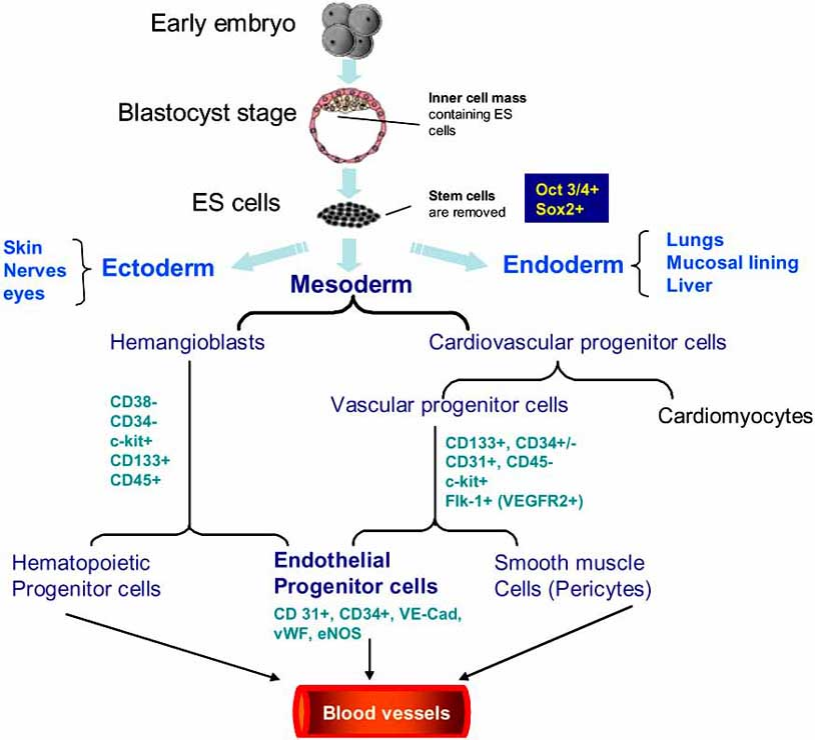
Development of vascular cells from human embryonic stem cells. Hemangioblasts are bipotential precursors to hematopoietic progenitor cells and endothelial progenitor cells (EPCs). The origins of smooth muscle cells (SMCs) are dependent on the location in the embryo. Vascular progenitor cells (VPCs) can further differentiate to mural cells (SMCs and pericytes) and endothelial cells. EPCs may trans-differentiate into SMCs during vessel formation. ES cells express Oct3/4, and Sox2; vascular progenitors express CD133, CD31, CD34, c-kit, and Flk-1 (VEGFR-2 receptor); and endothelial progenitors express CD31, CD 34, VE-Cadherin, vWF, and eNOS.

Life is regenerative. But by and large, humans lack the regenerative capacity of creatures such as newts and hydra. Emerging interest in using endothelial cells for therapeutic purposes has led to assessing hemodynamic forces as biomechanical stimuli for enriching embryonic stem (ES) cell commitment to endothelial progenitor cells. Endothelial cells (ECs) are critical cellular components of blood vessels. The denudation of the intact endothelial monolayer can cause lipid accumulation, monocyte adhesion, and inflammatory responses that initiate atherogenesis [1]. Pluripotent ES cells are capable of differentiating into all cell including neurons, cardiomyocytes, hematopoeitic cells, endothelial cells, osteogenic cells, and chondrocytes (Fig. **1**) [2-5]. 

## BIOMECHANICAL FORCES AND VASCULAR BIOLOGY

**Fig. (2):**
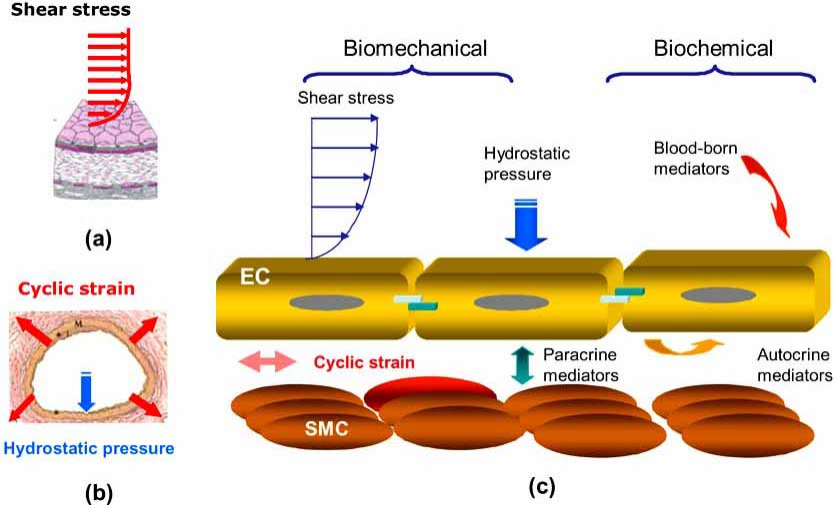
Schematic diagram of hemodynamic forces acting on endothelial cells (EC) and smooth muscle cells (SMC) in the blood vessel wall. (a) Fluid shear stress, the tangential frictional force by virtue of blood viscosity, acts on ECs. (b) Cyclic strain exerts a circumferential stretch on arterial wall in response to cardiac contraction. Hydrostatic pressure acts perpendicularly on ECs. (c) ECs are constantly exposed to both biomechanical and biochemical stimuli, which modulate endothelial functional phenotype. The biochemical stimuli include hormones, growth factors, cytokines, and bacterial products that can be delivered *via* the blood or *via* autocrine or paracrine mechanisms [7].

Vascular endothelial cells in the resistant arteries are constantly exposed to the dynamic changes of blood flow (Fig. **2**). The hemodynamic forces can be resolved into three components: (1) shear stress, the tangential frictional force acting at the endothelial cell surface, (2) hydrostatic pressure, the perpendicular force acting on the vascular wall, and (3) cyclic strain, the circumferential stretch of vessel wall [6].

In response to biomechanical forces, namely, circumferential stretch of arterial wall and fluid shear stress, vascular ECs undergo elongation in morphology in alignment with the direction of blood flow. In response to shear stress, ECs respond metabolically by altering the pro duction of vasodilating substances, including nitric oxide, prostacyclin, C-type natriuretic peptide, and adrenomedulin [8]. These fluid flow-induced phenotypic changes in EC function are often accompanied by genotypic changes in the expression of related genes [9]. 

**Fig. (3):**
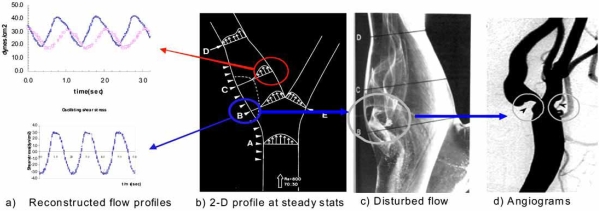
Shear stress and atherosclerosis. (a) Pulsatile versus oscillatory flow profiles at the reattachment point and the medial wall of bifurcation. (b) Pulsatile flow (red) occurs at the medial wall, whereas oscillating flow occurs at the reattachment point (blue). (c) The reattachment point is the site at which flow separation and disturbed flow occur. (d) Arterial angiogram shows plaque formation at the sites of flow separation. (e) CFD simulation. At the reattachment point, the mean shear stress is known to be near zero [10].

Shear stress imparts metabolic as well as mechanical effects on vascular endothelial function, and is intimately involved in angiogenesis and atherosclerosis [1, 8, 11]. During atherosclerosis, disturbed flow, including oscillatory flow (bidirectional net zero forward flow), is considered to be an atherogenic hemodynamics, developing at the lateral wall (Fig. **3**). By comparison, pulsatile flow is atheroprotective, developing at the medial wall of bifurcation or straight segments, and is responsible for down-regulating adhesion molecules, inflammatory cytokines, and oxidative stress [12-14]. 

## STEM CELL, FLUID FLOW, AND VASCULOGENESIS

ES cell lines established from the inner cell mass of blastocysts have the potential to differentiate into all embryonic cell lineages (Fig. **1**) [15]. Developmentally, hemodynamic forces play an important role during myocardiogenesis. Intracardial fluid shear stress is an essential epigenetic factor for zebra fish embryonic cardiogenesis, and absence of fluid flow results in the formation of an abnormal cardiac chambers and valve formation [16]. Disturbed flow is also a critical stimulus for morphologic embryonic heart. The direction of fluid flow on the node of mouse embryos determines the left-right asymmetry in the body plan [17].

Fluid flow also influences the differentiation of ES cells [18, 19] and mesenchymal progenitors [20] to vascular endothelial cells. Yamamoto *et al.* reported that fluid flow affected differentiation of human bone marrow-derived endothelial progenitor cells (EPCs) to ECs [21]. The molecular mechanisms by which hemodynamic forces regulate ES cell differentiation to vascular progenitor cells are an intense area of research for restoring endothelial dysfunction, inducing angiogenesis, and forming blood vessel networks. 

## *IN VITRO* MODEL TO STUDY STEM CELL DIFFERENTIATIONS

**Fig. (4):**
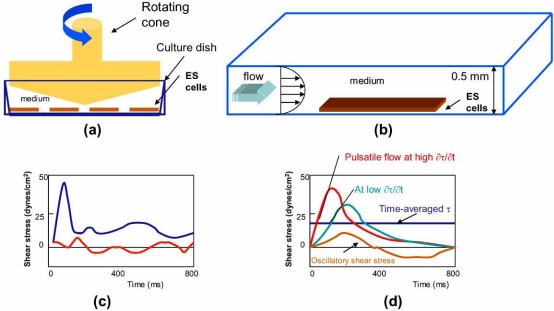
**(a)** Schematic diagram of the embryonic cells (ES) in a dynamic model. The cone-and-plate flow device was modified to optimize ES cells exposure to well-defined shear stress and confluent vascular progenitor monolayers were analyzed for endothelial cell markers. **(b)** Schematic diagram of the modified parallel plate model that was mounted on an inverted microscope. Confluent ES monolayers were monitored in response to specific shear stress patterns. **(c)** Representative hemodynamic flow profiles generated by the cone-and plate-system. Pulsatile shear stress is denoted in blue and oscillatory shear stress in red. **(d)** In the parallel-plate system, unidirectional laminar shear stress is denoted in dark blue, pulsatile shear stress at a high slew rate (∂τ/∂t) is denoted in red, low slew rate in green, and oscillatory shear stress in brown. Diagrams are not drawn to scale.

Developing new scale-up and cell separation technologies is critical to address the road blocks in basic stem cell research for clinical applications. Hemodynamic forces envisage the application of fluid flow in engineering *in vitro* production of differentiated cardiovascular cells. Using a modified cone-and-plate flow device, Blackman *et al.* simulated pulsatile shear stress in the common carotid artery and oscillatory shear stress in the lateral wall or point of flow separation in the internal carotid artery (Figs. **4a** and **4c**) [22]. Using the parallel plate flow system, Yamamoto *et al*. subjected the ES cells to laminar shear stress (Figs. **4b** and **4d**) [21]. The cone-and-plate model facilitated the assessment of embroid bodies (EBs) and bone marrow-derived endothelial progenitor cells in a confined volume [22]. The parallel-plate model elucidated new insights into the molecular mechanisms whereby variations in shear stress parameters influenced the rate and yield of CD31^+^ cells [21]. 

Numerous bioreactors have been developed in an attempt for isolation and purification of vascular progenitors. The precise control of local flow milieu has allowed for assessing bone marrow-derived EPCs homing onto the vascular endothelial cells [21]. The incorporation of feedback control system to regulate both biochemical and biomechanical parameters have provided insights into new scale-up and cell separation technologies. However, the focus remains to enrich the yield and rate CD31^+^ cell population that are capable of LDL uptake and tube-like formation in Matrigel [18, 21].

## BONE MARROW-DERIVED HEMATOPOIETIC STEM CELLS

Bone marrow cells contribute to the pathogenesis of vascular diseases in models of postangioplasty restenosis, graft vasculopathy, and hyperlipidemia-induced atherosclerosis [23]. It was suggested that bone marrow cells or hematopoietic stem cells (HSCs) may have the potential to give rise to vascular progenitor cells that home in on the damaged vessels and differentiate them into smooth muscle cells or endothelial cells [23]. During this process, incorporated bone marrow-derived EPCs are exposed to shear stress. Thus, fluid shear stress and local flow patterns may influence reendothelialization *via* EPC homing, proliferation, and differentiation. However, the yield of isolated EPCs remains low and the underlying mechanism remains unknown.

## EMBRYONIC STEM CELLS-DERIVED VASCULAR PROGENITOR CELLS

Human ES cells have been isolated to form stable pluripotent cell lines that are capable of unlimited proliferation under specific culture conditions. Human ES cells aggregate into clusters of cells or embroid bodies (EBs) that differentiate into multiple tissue lineages (Fig. **1**) [24-26]. These cells also appear to be weakly immunogenic, expressing moderate amounts of major histocompatibility complex (MHC) class I without MHC class II protein [27]. Multiple markers have been used to characterize the vascular-endothelial differentiation capabilities of human ES cells (Fig. **1**). Expression of vascular endothelial cadherin (VE-cad), platelet endothelial cell adhesion molecule-1 (PECAM-1), CD34, and Flk-1 (human part JDR, vascular endothelial growth factor receptor 2) and the ability to take up Dil-labeled acetylated low-density lipoprotein (Dil-Ac-LDL) have been used as markers for identifying endothelial precursors [28]. Mature ECs were identified by selective staining for von Willebrand factor (vWF), endothelial nitric oxide synthase (eNOS), and E-selectin proteins [29, 30]. Current road blocks in vascular stem cell research remain to develop new scale-up and cell separation technologies to deliver vascular progenitor cell population in high purity and stability with full retention of function.

## ENGINEERING ES CELL DIFFERENTIATION TO VPCS

Two main approaches have been used for purifying progenitor ECs from human ES cells under static *in vitro* conditions: (1) supplementing feeder layers for specific cell-surface molecules; and (2) selecting EBs for specific cell-surface molecules. The former used endothelial progenitors derived from EBs, undifferentiated hESC’s grown on various feeder layers from bone marrow stromal cells (S17 cell line) or mouse yolk-sac ECs (C166 cell line) [29]. After 17 days, undifferentiated human ES cells differentiated into an early hematopoietic subpopulation of CD34^+^CD31^-^CD45^-^ cells, with 50% of the CD34^+^ cells coexpressing PECAM-1 [29]. Under pituitary extract and vascular endothelial growth factor (VEGF), these cells became attached and spindled-shaped, strongly expressing PECAM-1, VE-cad, and capable of Dil-Ac-LDL uptake. The latter approach is based on isolating EBs by fluorescence-activated cell sorting (FACS) of PECAM-1^+^ cells that express mature endothelial protein vWF in addition to expression of CD34, Flk-1, and VE-cad, and being capable of Dil-Ac-LDL uptake [31]. After 7 to 12 days of differentiation, human EBs developed into adhesive and non-adhesive cells. Some adhesive cells were found to express PECAM-1 (50%) and VE-cad (11%), whereas CD45 cells (a marker for hematopoeitic progenitor cells) were not capable of taking of Dil-Ac-LDL [32]. Seeding these cells on Matrigel and supplementing with large amount of VEGF (50 μg/mL) resulted in a typical tubelike arrangement of elongated ECs within the matrix [33].

Recently, a novel method was developed to induce selective differentiation of ES cells into both vascular endothelial cells and mural cells (pericytes and vascular smooth muscle cells) [34, 35]. In this method, undifferentiated ES cells were cultured on type IV collagen-coated dishes, and VEGF receptor 2 (VEGF-R2) and Flk-1-positive (Flk-1^+^) cells were purified by flow cytometry sorting. The addition of VEGF to the cultures promoted endothelial differentiation, whereas mural cells were induced by platelet-derived growth factor (PDGF)-BB. The vascular cells derived from Flk1^+^ cells organized into vessel-like structures in 3-D culture and contributed to the developing vasculature *in vivo* [36]. Bai *et al*. chose CD34 as a marker to isolate human ES cell-derived endothelial progenitor cells [37]. CD34^+^ cells are not expressed in undifferentiated human ES cells that expressed VEGFR2 and CD133. CD34^+^ population was increased to approximately 10% at around day 12-15 from human ES cells by changing the differentiation medium of the mouse embryonic fibroblast (MEF) feeder cells to a serum free-medium in the presence of VEGF, FGF-2, and BMP-4 [31, 38, 39]. The isolated CD34^+^ progenitor cells by MACS column gave rise to cells with endothelial morphology and expressed endothelial cell markers CD31, VE-cad, and vWF in endothelial cell culture medium [38, 39]. Collaborative research will likely advance the vascular biology community to engineer rapid isolation of pure and stable vascular progenitors (CD31, VE-cad, and vWF cell population) with full retention of function.

## SHEAR STRESS AND ES CELL COMMITMENT TO VPCS

Proliferation and differentiation of ES cells are promoted not only by "chemical stimuli" such as VEGF, PDGF, and TGF-β [34, 35, 40, 41], but also by fluid shear stress. Laminar shear stress at 10 dyn/cm^2 ^activated transcription from VEGF-R2 promoter [42]. Furthermore, shear stress at 1.5 to 10.0 dyn/cm^2 ^increased the cell density of mouse Flk-1^+^ or VEGF-R2^+^ cells [35]. Cell cycle analysis demonstrated that shear stress decreased a larger percentage of the cells in the G_0_ and G_1_ phase and increased the percentage of cells in the S and G_2_-M phases in comparison with Flk-1^+^ ES cells cultured under static conditions. Shear stress also increased the expression of the vascular endothelial cell-specific markers Flk-1, Flt-1, VE-cad, and PECAM-1, but it had no effect on expression of the mural cell marker smooth muscle α-actin, blood cell marker CD3, or the epithelial cell marker keratin [18].

Zheng *et al.* reported that exposing ES cell-derived ECs (defined as stem cell antigen-1-positive or Sca-1+) to shear stress at 12 dyne/cm^2^ for 4 days increased the proliferation by ~70% [43]. RT-PCR analysis revealed that withdrawal of LIF and culture on collagen IV-coated slides increased mRNA expression of PECAM-1 (CD31), prominin 1 (CD133), VE-cad(CD144), Flt-1, and Flk-1 in Sca-l^+^ progenitor cells [43]. Moreover, shear stress was implicated in up-regulation of the transcription factor myocyte enhancer factor-2 (MEF-2C) in ES cells. MEF-2C is highly important for cardiovascular development. Formation of MEF-2C-Smad4/CBP complexes was observed in shear stress-treated ES cells, recapitulating some events occurring during ES cell differentiation into cardiovascular precursors [42, 44, 45]. These findings indicate that shear stress selectively promotes the differentiation of Flk-1^+^ ES cells into the endothelial cell lineage to form tubular network significantly faster than the static controls [35].

## SHEAR STRESS AND EPIGENETIC MODIFICATION OF HISTONES

During embryonal organogenesis, chromatin remodeling plays an important role in regulating differentiation [46]. Shear stress promotes decondensation of chromatin to allow gene transcription. Epigenetic modification of histones plays an important role in chromatin condensation/decondensation [42]. Histones can be acetylated, phosphorylated, and methylated by distinct classes of enzymes, namely histone acetyltransferases (HATs), histone deacetylase (HADACs), and histone methyltransferases. 

Shear stress regulates gene expression by inducing epigenetic modification of histones and influences cell differentiation in mouse ES cells. Zeng *et al. *showed that laminar flow activated histone deacetylase 3 (HDAC3) through the Flk-1-PI3K-Akt pathway and that HDAC3-mediated p53 deacetylation and p21 activation were crucial for shear stress and VEFG-induced EC differentiation [43]. Illi *et al.* found that histone H3 phphorylation on serine 10 (S10) occurred within 30 minutes of shear stress exposure at 10 dyn/cm^2 ^[42]. Hence, fluid shear stress plays an important role in enriching and accelerating ES-derived vascular progenitor cells.

## VASCULAR PROGENITOR CELLS AND IMPLICATION FOR CARDIOVASCULAR MEDICINE

ES cells are advantageous for cell transplantation to repair damaged ischemic tissues and restore endothelial dysfunction by virtue of their high proliferation capacity and pluripotency. ES cells undergo spontaneous *in vitro* differentiation, leading to the formation of cardiovascular precursors among other cell types [5, 29, 47]. This process normally occurs in several days. However, exposure to shear stress accelerates the onset of cell expressing cardiovascular markers that become detectable after 24 hour of flow exposure. However, challenges remain to address the restorative capacity, survival, engraftment, and tumorigenicity of human ES cell-derived endothelial cells. While a vast majority of research concerning EC differentiation from ES cells is derived from experiments involving growth factors and hypoxia environment [42], novel applications of local biomechanical milieu has provided an entry point to engineer artificial vessels to repair damaged vessels and to form vessel networks. 

## SUMMARY

Generation of endothelial cells from human ES cells not only provides a ready cell resource for potential clinical applications, but also paves an excellent avenue to study vasculogenesis and angiongenesis events in the human system. The endothelial progenitors isolated from human ES cells have various phenotypes because of varying derivation protocols and the supplementation of growth factors; even more importantly, they were isolated at different stages of development [29]. Critical questions remain to address the road blocks in basic stem cell research for clinical applications: (1) understanding the kinetics of ES cell differentiation into VPCs, (2) the molecular mechanisms whereby shear stress induce ES differentiation to VPCs, (3) stability and function of ES cell-derived VPCs in formation of durable blood vessels. The recent California Institute for Regenerative Medicine Tools and Technologies Awards (RFA08-02) have garnered momentum to develop a scale-up and cell purification system that will interface delivery of purified progenitors with molecular imaging to track stem cell function in the injured tissue function. The recent NHLBI Progenitor Cell Biology Consortium Planning Awards (RFA-HL-08-012) has further synergized multi-disciplinary efforts to identify and characterize progenitor cell lineages, to direct the differentiation of stem and progenitor cells to the desired cell fates, and to develop new strategies to address the unique challenges presented by the transplantation of these cells. 

## ABBREVIATIONS

EPC: = Endothelial progenitor cell
ES cells: = Embryonic stem cells
EB: = Embryonic body
ECs: = Endothelial cells
VPC: = Vascular prognitor cells
HSC: = Hematopoietic progenitor cells
